# Items

**Published:** 1910-07

**Authors:** 


### ITEMS.

Schering & Glatz, for the past forty-three years have been located at No. 58 Maiden Lane, New York, but on July 1, moved into a new home at 150-152 Maiden Lane, only a few blocks from their former location.

The Leighton Wright Company, Buffalo, has recently opened an especially attractive wholesale and retail store at No. 46 West Chippewa Street. This corporation will deal in medical supplies, surgical instruments and rubber goods and has opened its store with a full line of all. The prescription department will be an especial feature of the business and the patronage of physicians in western New York is solicited. Complete satisfaction is guaranteed. None of the so-called patent preparations will be carried in stock. An advertisement appears on another page of the Journal, to which attention is invited.

Walter S. McEwan, formerly of Albany, N. Y., has purchased an interest in, and taken over the active management of the Radu Surgical Instrument Co., makers of high grade electrically illuminated diagnostic cases, high frequency and $x$-ray machines, of North Tonawanda, N. Y. The change contemplates new factories and the general extension of an already prosperous business.

